# Association of Habitual Dietary Intake with Liver Iron—A Population-Based Imaging Study

**DOI:** 10.3390/nu14010132

**Published:** 2021-12-28

**Authors:** Jule Filler, Ricarda von Krüchten, Nina Wawro, Lisa Maier, Roberto Lorbeer, Johanna Nattenmüller, Barbara Thorand, Fabian Bamberg, Annette Peters, Christopher L. Schlett, Jakob Linseisen, Susanne Rospleszcz

**Affiliations:** 1 Institute of Epidemiology, Helmholtz Zentrum München, German Research Center for Environmental Health, Ingolstädter Landstraße 1, 85764 Neuherberg, Germany; jule.filler@yahoo.com (J.F.); lisamaier2309@gmail.com (L.M.); thorand@helmholtz-muenchen.de (B.T.); peters@helmholtz-muenchen.de (A.P.); 2 Institute for Medical Information Processing, Biometry and Epidemiology—IBE, LMU Munich, 81377 Munich, Germany; j.linseisen@unika-t.de; 3 Pettenkofer School of Public Health, LMU Munich, 81377 Munich, Germany; 4 Department of Diagnostic and Interventional Radiology, Medical Center, University Freiburg, 79106 Freiburg, Germany; ricarda.kruechten@uniklinik-freiburg.de (R.v.K.); johannanattenmueller@gmail.com (J.N.); fabian.bamberg@uniklinik-freiburg.de (F.B.); christopher.schlett@uniklinik-freiburg.de (C.L.S.); 5 Independent Research Group Clinical Epidemiology, Helmholtz Zentrum München, German Research Center for Environmental Health (GmbH), 85764 Neuherberg, Germany; nina.wawro@helmholtz-muenchen.de; 6 Chair of Epidemiology, University Augsburg at University Hospital Augsburg, 86156 Augsburg, Germany; 7 Department of Radiology, University Hospital, LMU Munich, 80336 Munich, Germany; roberto.lorbeer@med.uni-muenchen.de; 8 German Centre for Cardiovascular Research (DZHK e.V.), Partner Site Munich Heart Alliance, 80802 Munich, Germany; 9 Clinic of Diagnostic and Interventional Radiology (DIR), Heidelberg University Hospital, 69120 Heidelberg, Germany; 10 German Center for Diabetes Research (DZD), München-Neuherberg, 85764 Neuherberg, Germany

**Keywords:** liver iron, diet, MRI, nutrition, iron metabolism, alcohol, liver iron overload, population-based

## Abstract

Iron-related disorders of the liver can result in serious health conditions, such as liver cirrhosis. Evidence on the role of modifiable lifestyle factors like nutrition in liver iron storage is lacking. Thus, we aimed to assess the association of habitual diet with liver iron content (LIC). We investigated 303 participants from the population-based KORA-MRI study who underwent whole-body magnetic resonance imaging (MRI). Dietary habits were evaluated using repeated 24 h food lists and a food frequency questionnaire. Sex-stratified multiple linear regression models were applied to quantify the association between nutrition variables of interest and LIC, adjusting for liver fat content (LFC), energy intake, and age. Mean age of participants was 56.4 ± 9.0 years and 44.2% were female. Mean LIC was 1.23 ± 0.12 mg/g dry weight, with higher values in men than in women (1.26 ± 0.13 and 1.20 ± 0.10 mg/g, *p* < 0.001). Alcohol intake was positively associated with LIC (men: β = 1.94; women: β = 4.98, *p*-values < 0.03). Significant negative associations with LIC were found for fiber (β = −5.61, *p* < 0.001) and potassium (β = −0.058, *p* = 0.034) for female participants only. Furthermore, LIC was highly correlated with liver fat content in both sexes. Our findings suggests that there are sex-specific associations of habitual dietary intake and LIC. Alcohol, fiber, and potassium may play a considerable role in liver iron metabolism.

## 1. Introduction

Dysregulation of iron metabolism, such as iron overload or deficiency, can lead to systemic iron-related disorders. Since there is no excretory pathway for iron in the mammalian metabolism, iron regulation consists of finely tuned, yet vulnerable processes to control the duodenal uptake of dietary iron and the systematic reuse of body iron. The liver is a major production site for many proteins of iron homeostasis. Furthermore, liver cells can store excess iron and release it into the bloodstream when needed [1]. In iron-related disorders, especially regarding iron overload, the liver is typically one of the most affected organs, exhibiting excess liver iron content (LIC) [2]. The two most common causes of iron overload are hereditary hemochromatosis and dysmetabolic iron overload syndrome (DIOS), a condition with multifactorial metabolic etiology. The mechanisms underlying DIOS have yet to be fully investigated. Individuals with obesity, alcoholic liver disease, type 2 diabetes, or chronic hepatitis C, present with DIOS in 15–50% of the cases [1]. Lifestyle factors like nutrition play a critical role in the development of non-congenital risk factors for DIOS. On the other hand, chronic liver disease associated with gastrointestinal bleeding can result in anemia and iron deficiency anemia [3]. One third of patients with non-alcoholic fatty liver disease have been reported to be iron deficient [4], which may also be associated with DIOS [5].

For the prevention of iron-linked pathological conditions it is, therefore, important to identify potentially modifiable risk factors for iron excess or deficiency, particularly in the liver. Nutrition and diet have been associated with markers of iron metabolism. In the Framingham Heart Study, whole-grain intake was associated with a decreased risk of high serum ferritin [6], which was confirmed by other studies [7,8]. Similar results have been reported for fiber consumption [9,10,11]. Other dietary components that have been found to be negatively associated with total body iron are, e.g., vegetables [7] and calcium [10,11]. In a Danish cohort, alcohol consumption was linked to higher levels of serum ferritin [12] and comparable findings have been reported recently [10,13,14]. Furthermore, the intake of total [10,11] and heme iron [8,11], red meat [8,15], and vitamin C [16,17] have been suggested to be positively related to levels of total body iron markers.

However, these aforementioned studies mainly used blood biomarkers of iron, such as serum ferritin or transferrin saturation. Levels of these blood markers depend not only on iron status but are also affected by inflammation, chronic alcohol abuse, and other metabolic conditions [18]. Thus, the validity of these markers as proxies for liver iron content (LIC) is questionable. Moreover, population-based studies on the association of LIC and nutrition are lacking. This is mainly due to the complexity of reliable LIC measurement on a larger scale. The gold standard for LIC quantification is liver biopsy, which is however unsuitable for a population-based sample. The most precise, non-invasive LIC determination method for population-based studies is Magnetic Resonance Imaging (MRI). Additional advantages of MRI-based LIC determination are its reliability and reproducibility [18].

Yet, comprehensive analyses of the relationship between nutrition and LIC remain to be reported. A single preliminary result from the UK Biobank [19] pointed at a possible association between beef intake and LIC. To our knowledge, no further research has been performed on nutrition and LIC. Therefore, in the present study, we exploratively aim to assess the association of habitual dietary intake with MRI-derived LIC in a well-characterized sample from a population-based study, the Cooperative Health Research in the Region of Augsburg (KORA).

## 2. Materials and Methods

### 2.1. Study Population

**Figure 1:**
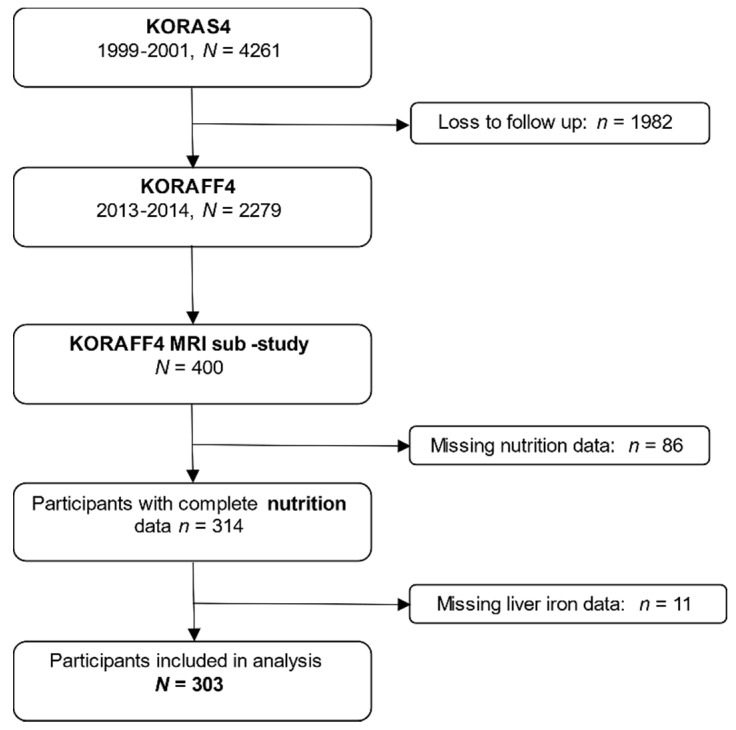
Participant flow chart. Among 400 individuals who underwent the MRI-examination, a total of 303 were included in the analysis.

Participants stem from the population-based KORA FF4 study (2013–2014, *N* = 2279), the second follow-up of the KORA S4 baseline study (1999–2001, *N* = 4261), which was sampled in the region auf Augsburg, Germany. Details on the general setup of the KORA studies are described elsewhere [20]. The present analysis included participants of an MRI sub-study in KORA FF4 who also completed a comprehensive dietary assessment. In the MRI sub-study, 400 participants without history of cardiovascular disease underwent whole-body MR imaging. Study design, exclusion criteria, and details of the whole-body imaging protocol of the KORA-MRI study are described elsewhere [21]. All participants were genotyped for SNP rs1800562, the major mutation of the HFE-gene related to hereditary hemochromatosis with the Affymetrix Axiom Chip [22]. In women, post-menopause was defined when they reported no menstrual bleeding during the previous 12 months, hysterectomy, or hormonal therapy inducing bleeding cessation. The study was conducted according to the Declaration of Helsinki and approved by the local ethics committee. Each participant provided written informed consent prior to study participation. Complete dietary data were available for 314 of the 400 participants. Eleven participants could not be included in the present analysis due to missing liver iron data. A detailed participant flowchart is depicted in Figure 1.

### 2.2. Assessment of Liver Iron Content with Magnetic Resonance Imaging

Whole-body imaging was performed on a 3-Tesla Magnetom Skyra (Siemens Healthcare, Erlangen, Germany). Liver iron content was ascertained using a high-speed T2-corrected multi-echo spectroscopy (HISTO) technique on a single-voxel spectroscopy sequence with stimulated-echo acquisition mode [23,24]. This sequence allows for a simultaneous estimation of liver fat content (LFC) as hepatic proton density fat fraction (PDFF) and liver iron content (LIC) through the relaxation rate R2 *. For the present analysis, liver iron was averaged over the left and right liver lobe. The unit of LIC was converted from s^−1^ into mg/g or µg/g dry weight using a previously specified formula [25]. Details and comparability to conversions with other formulas are described in Table S1. The conversion from s^−1^ to µg/g dry weight was done to improve interpretability of the unit and to enable comparison with other studies.

### 2.3. Assessment of Habitual Dietary Intake

Usual food and nutrient intake were estimated based on a blended approach, combining repeated 24-h food lists (24H-FL) and a food frequency questionnaire (FFQ). Up to three 24H-FL per participant were used to assess which foods had been consumed over the previous day and consisted of 246 food items. FFQs queried the consumption frequency of 148 items to estimate dietary habits in the past 12 months. Usual daily intake of food items was estimated for each participant by combining estimated consumption probability and amount. Consumption probability was estimated by logistic mixed models adjusted for covariates and FFQ information. Consumption amount was estimated based on the second Bavarian Food Consumption Survey (BVS II). Usual intake estimates were summarized into 16 food groups and 21 subgroups based on the EPIC-Soft classification scheme. Energy and nutrient intakes were estimated by means of the German food composition table BLS, version 3.02. Dietary intake data were available for a total of 1602 participants from KORA FF4. Further details are delineated elsewhere [26]. The choice of nutrition variables analyzed in the present explorative study was guided by previous literature. The present analysis includes macronutrients (carbohydrates, protein, fat, and alcohol) as well as related subcategories (fiber, sugar, and fatty acids) and selected micronutrients (sodium, potassium, calcium, magnesium, phosphorus, chloride, iron, and zinc; and vitamins A and C). Moreover, the food groups meat, cereal products, whole grain products, vegetables, fruits, and dairy products were included in the analysis.

### 2.4. Statistical Analysis

The study sample characteristics are described as arithmetic means and standard deviations for continuous and as counts and percentages for categorical variables. LIC, LFC and nutrition variables are presented as arithmetic means and standard deviations. LFC is additionally described by median and interquartile range due to its skewed distribution. Distribution of data was checked visually by inspecting histograms and density plots. Differences in continuous and categorical variables between men and women were examined by *t*-test and χ^2^-test, respectively. In women, LIC was additionally assessed by menopausal status and compared using *t*-test. All statistical analyses were stratified by sex. Correlations between LIC and study sample characteristics were calculated by Spearman’s Rho. Multiple linear regression models were calculated to assess the association between continuous LIC and each continuous nutrition variable of interest. Each model was adjusted for energy intake, age, and log-transformed LFC. In this explorative analysis, *p*-values < 0.05 were considered to indicate statistical significance. All statistical analyses were performed using R version 3.6.1.

## 3. Results

### 3.1. Study Sample

A final sample of 303 participants was included in the analysis. Baseline characteristics of the whole study sample, women (*n* = 134, 44.2%) and men (*n* = 169, 55.8%), are provided in Table 1. Baseline characteristics for women according to menopausal status are shown in Supplementary Table S2. Participants were middle-aged with a mean age of 56.4 (± 9.0) years, more than a third (35.3%) had hypertension, and 12% had diabetes. Men were more likely to have hypertension, type 2 diabetes, or prediabetes, and be physically inactive than women. BMI was not significantly different between sexes. HFE-genotyping revealed that no participant had a homozygous mutation in r11800562. The 97 excluded participants did not differ with respect to LIC, LFC, and demographic characteristics (Table S3). Mean LIC was 1.26 mg/g (SD = 0.13 mg/g) in men and 1.20 mg/g (SD = 0.10 mg/g) in women (*p* < 0.001). The maximum LIC values that were observed in the study sample were 1.71 mg/g for men and 1.5 mg/g for women. Male participants also had higher LFC than women (10.35%, SD = 8.4%, and 6.33%, SD = 6.4%, respectively, *p* < 0.001).

### 3.2. Habitual Food and Nutrient Intake

The distribution of *usual food and nutrient intake* is presented in Table 2. Mean daily energy intake was 1840 kcal/d, and men had a higher mean energy intake than women. On average, men consumed higher amounts of carbohydrates, fats, protein, and alcohol than women. Fiber intake was only slightly higher in men than in women. Furthermore, all micronutrient intake was higher in men than in women, except for vitamin C intake, which was higher in women. Female participants consumed lower amounts of meat but higher quantities of vegetables, fruits, and dairy products than men. For most nutritional factors, differences in habitual intake between men and women were statistically different (Table 2).

### 3.3. Association of Baseline Characteristics with Liver Iron Content

**Figure 2:**
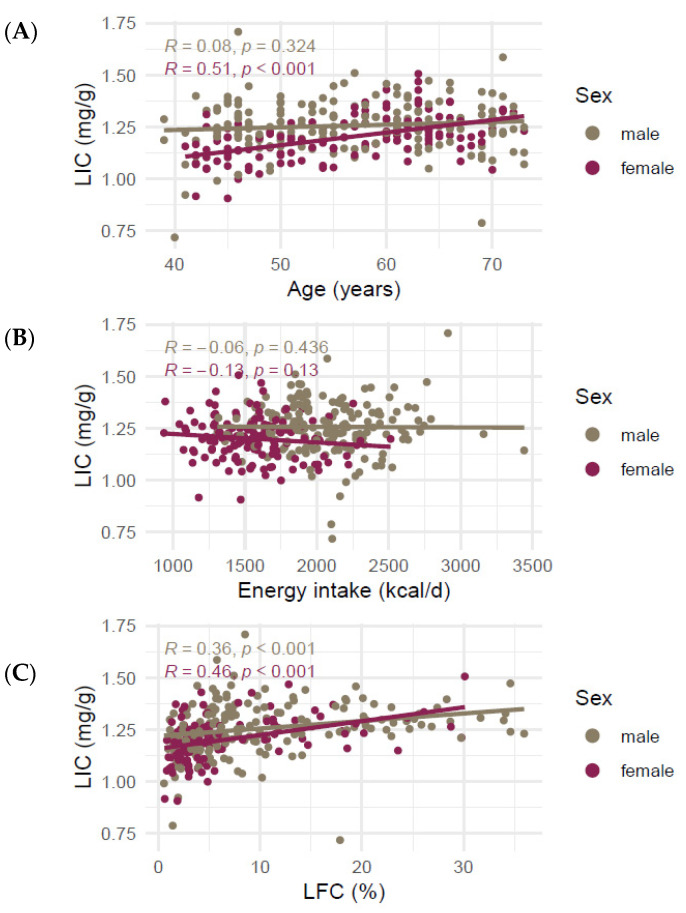
Correlation of liver iron content in mg/g and (**A**) age, (**B**) mean daily energy intake, and (**C**) liver fat. Lines represent lines of best fit in a univariate linear model. R denotes Spearman’s Rho. Abbreviations: LIC liver iron content, LFC liver fat content.

The correlation of age with LIC was very different between the sexes. While age was not correlated with LIC in male participants (R = 0.094, *p* = 0.22), it correlated strongly with LIC in female participants (R = 0.51, *p* < 0.001, Figure 2A). LIC was significantly different between pre- and postmenopausal women (1.13 ± 0.09 mg/g and 1.23 ± 0.09 mg/g, respectively, *p* < 0.001, Figure S1). Liver iron was not significantly correlated with mean daily energy intake (Figure 2B), LFC was positively correlated with LIC for both sexes (R = 0.36 (men) and R = 0.46 (women), *p*-values < 0.001, Figure 2C).

### 3.4. Association of Macronutrients with Liver Iron Content

For men, multiple linear regression models yielded no significant association between LIC and carbohydrate, protein, and fat intake. Higher alcohol consumption was significantly associated with higher LIC (β = 1.698, *p* = 0.050). In women, higher carbohydrate intake was marginally significantly associated with lower LIC (β = −0.970, *p* = 0.063), while there was no association for protein and fat consumption. Like in men, higher alcohol intake was significantly associated with increased LIC (β = 5.615, *p* < 0.001) in women. Further analyses showed no significant association between fiber intake and LIC for men, but a significant negative association for women (β = −5.818, *p* = 0.023). Total sugar intake and all five types of the investigated fatty acids were not associated with LIC in men or women (Table 3).

### 3.5. Association of Selected Micronutrients with Liver Iron Content

None of the models for the micronutrients of interest, including the trace element iron, yielded a significant association with LIC in men. In women, there was a significant negative association between potassium intake and LIC (β = −0.058, *p* = 0.034) and a marginally significant negative association between magnesium intake and LIC (β = −0.505, *p* = 0.071) (Table 3).

### 3.6. Association of Selected Food Groups with Liver Iron Content

For both men and women, meat or red meat intake was not significantly associated with LIC. The same lack of association was observed for cereals as well as whole grain products. Vegetable and fruit intake were not associated with LIC for women; however, higher vegetable intake was marginally significantly associated with higher LIC in men (β = 0.420, *p* = 0.056). Fruit consumption was not associated with LIC in men either in combination with vegetable intake, or independently. Dairy product intake was not significantly associated with LIC in men or women (Table 3).

## 4. Discussion

This study used cross-sectional data on LIC and habitual diet to exploratively investigate sex-specific associations between nutrition and MRI-derived liver iron. We observed that LIC was elevated in individuals with higher alcohol consumption. In women, a higher intake of fiber, carbohydrate, potassium, and magnesium was associated with lower LIC. Total iron intake and meat consumption were not associated with LIC.

### 4.1. Distribution of Liver Iron and Correlation with Age

In the present study sample, LIC was distributed similarly as in two population-based studies from Germany [27] and the UK [19] (medians: 1.08 and 1.25 as compared to ours with 1.23 mg/g). No individual in our study exceeded a threshold of 1.8 mg/g for liver iron overload [19]. Male participants had higher LIC than women, confirming previous findings [19,27,28]. In women, age correlated significantly with LIC. Even though the UK Biobank study did not show such an effect [19], comparable associations in women between age and LIC [27] and age and serum ferritin [29,30] have been found before. In our sample, postmenopausal women had significantly higher LIC than premenopausal women. The onset of menopause is most likely the mechanism related to the observed age effect, due to the cessation of iron loss from regular bleeding. Osler et al. [12] found a relationship between menopausal status and serum ferritin in women. Confirmingly, age accounted for about 20% and less than 0.5% of LIC variability in female versus male participants, respectively.

### 4.2. Relationship of Liver Fat and Liver Iron

LIC correlated significantly with LFC for both sexes, which is in line with earlier reports [19,27]. Our results suggest that LFC accounts for approximately 5% of LIC variability in men and 10–15% in women (as calculated by adjusted R^2^, see Table 3). Previous findings suggest a considerable interplay of obesity and visceral adipose tissue and iron metabolism [31]. Furthermore, iron plays a role in the severity of metabolic syndrome [5] and non-alcoholic fatty liver disease (NAFLD) [32]. Higher liver iron stores contribute to oxidative stress, a central factor in the pathogenesis of non-alcoholic steatohepatitis, fibrogenesis, and inflammatory mechanisms. Adipose tissue iron has been linked to insulin resistance, another pathway through which iron may be involved in NAFLD pathogenesis [32]. Our results therefore confirm previous findings indicating the relationship between hepatic adipose tissue and iron concentration. In addition, our results suggest that this association may be more pronounced in women than in men.

### 4.3. Association of Alcohol with Liver Iron

This study revealed a significant positive association between daily alcohol intake and LIC. Models including alcohol as a covariate provided the highest explanatory power (Adjusted R^2^ ≈ 0.4 for women and ≈0.11 for men). In our sample, there were no participants with zero alcohol intake, which could be due to genuine alcohol abstinence or due to the algorithm to calculate habitual diet.

Previous studies have established associations between alcohol intake and markers of iron status [12,13,14,16]. A possible mechanism by which ethanol interferes with liver iron metabolism is via suppression of the peptide hormone hepcidin produced by the hepatocytes. Several previous studies have found decreased hepcidin levels in patients with alcoholic liver disease [33,34]. Hepcidin is one of the most important iron homeostasis regulators and is increased in the presence of higher iron levels in the system, inducing signaling pathways that result in down-regulation of iron absorption in the duodenum [1,35]. Alcohol has been proposed to suppress hepcidin expression [36,37,38].

Alcohol appears to affect body iron stores already at a consumption of smaller amounts. For example, Ioannou and colleagues [13] reported a 40% risk reduction of iron deficiency anemia for any alcohol consumption, as well as an increase in the risk of iron overload in participants who consumed more than two alcoholic drinks per day. In another study, iron markers were significantly increased, even in individuals with non-harmful alcohol consumption [14].

Therefore, our results confirm and extend these previous findings in demonstrating a positive association of dietary alcohol with LIC, independent of liver fat. As one of our major findings, the association between alcohol and LIC requires further investigation in larger study samples, as well as regards the effects of different dietary sources of alcohol.

### 4.4. Association of Fiber, Carbohydrates, and Vegetables with Liver Iron

We found negative associations between fiber and carbohydrate intake and LIC in women. Grain and fiber consumption have been linked to lower total body iron stores previously [7,8,11]. Fiber is rich in phytates, a potent inhibitor of iron absorption in the intestines [39,40,41]. A reason for the sex difference could be that relative to the total energy intake, women in our sample consumed more dietary fiber than men. In men, vegetable consumption was associated with higher LIC. We take this result with caution, given that it was only marginally significant and our findings did not suggest an association between fiber and LIC in men. One possibility is that this is a false-positive result, which is always possible given the exploratory nature of our study. Second, the vegetable intake in men could be correlated with other food items which we did not evaluate that could be associated with increased liver iron. Either way, the direction of association would contradict previous findings regarding serum ferritin and hemoglobin levels [7,42]. Even though vegetables, especially leafy green vegetables, are a source of non-heme iron, they also contain high levels of secondary phytochemicals like polyphenols and phytates, which can significantly reduce iron bioavailability [40,43]. Hence, our findings on the intake of fiber-rich foods confirm previous results, but only for women and not for men. We can only speculate about the reasons for this. Regarding the lack of significance of carbohydrates and fiber, we hypothesize that the effect in men might be smaller compared to the effect in women, and thus we were unable to find a statistically significant estimate—this is supported by the fact that the effect direction is the same in men compared to women. Taken together, more research is needed to particularly assess sex-specific effects of fiber intake on iron storage.

### 4.5. Association of Potassium and Magnesium with Liver Iron

Our results also indicate a negative association between the intake of the minerals potassium and magnesium with LIC for women. Potassium and magnesium intake were highly correlated in both sexes (Pearson R > 0.9, *p* < 0.001, respectively). A meta-analysis by Cai et al. [44] found that potassium intake is generally associated with lower risk of metabolic syndrome (MetS). The meta-analysis did not evaluate sex-specific differences; however, one study found the association between low potassium intake and increased prevalence of MetS only for women [45]. Links between serum magnesium or dietary magnesium intake with hepatic fat and MetS have also been reported: Higher magnesium intake was found to be associated with lower odds of fatty liver disease and prediabetes [46]. A systematic review from 2016 [47] concluded that oral magnesium supplementation might reduce MetS burden in individuals with low magnesium stores. The available research does not explicitly hint at a sex-specific relationship of potassium and magnesium with MetS and related disorders [44]. Nevertheless, it is possible that the association between these minerals and LIC is more pronounced in women than in men, which may explain the differing results of our analysis. The present study, therefore, extends previous findings as far as to suggest an association of dietary potassium and magnesium not only with LFC but also independently with LIC.

### 4.6. Intestinal Iron Bioavailability

Findings from the UK Biobank indicated an association between beef consumption and LIC [19]. In our study, we did not find an association between red meat and LIC or between total iron intake and LIC, suggesting that intra-cellular hepatic iron concentration does not reflect the absolute amount of iron consumption. This could be explained by extensive regulatory mechanisms such as duodenal bioavailability. Aside from phytates and polyphenols, the literature suggests other foods and nutrients that potentially inhibit duodenal iron absorption. These include calcium [48,49] and peptides from proteins [41,50]. On the contrary, ascorbic acid (vitamin C) [51] and muscle and liver tissue from animal sources [52] constitute proposed enhancers of iron bioavailability. Furthermore, duodenal iron uptake is reduced in DIOS, again suggesting a relationship with increased liver fat [53].

### 4.7. Strengths and Limitations

This study has limitations. Its explorative nature implies no claim for implicit generalizability of the results to broader or specific populations. Firstly, the sample for which nutrition and LIC data were available consisted of 303 participants, which attenuates statistical power. Keeping in mind that total body MRI is a rather elaborate assessment method, the sample size can be considered reasonable for the aim of hypothesis generation. Secondly, critical intermediate variables, such as estimated bioavailable iron or blood-based markers, like serum ferritin, were not available and should be incorporated in future analyses. Moreover, sex-specific interaction effects of metabolic and nutrition factors, as well as important genetic effects beyond the presence of r11800562 are other parameters possibly contributing to LIC. Consequently, further efforts in larger studies are advisable to corroborate the present findings.

Nevertheless, our study presents a number of strengths. These include its well-characterized study sample from an underlying population-based cohort. This is particularly relevant, as most previous studies on liver iron worked with patient-based data. In addition, LIC was precisely measured with MRI, and habitual dietary intake was assessed with a reliable blended approach that combined 24H-FLs and an FFQ. Thus, our results provide a rich foundation for future efforts to characterize the effects of nutrition on liver iron status.

## 5. Conclusions

Our results suggest a relationship between diet and LIC. Dietary alcohol, fiber, and the minerals magnesium and potassium might be involved in mechanisms of liver iron homeostasis, irrespective of the amount of hepatic adipose tissue. In particular, the results point to iron bioavailability and absorption as potential key factors influenced by nutrition, implying that the role of nutritional factors goes beyond their mere iron content. Future work should focus on the disentanglement of the role of diet in liver iron metabolism and should take blood-based biomarkers and iron bioavailability into account.

## Figures and Tables

**Table 1:** Baseline characteristics of the study population.

	All	Men	Women	
	*N* = 303	*N* = 169 (55.8%)	*N* = 134 (44.2%)	*p*-value Men vs. Women
Age (years) Menopausal status premenopausal postmenopausal	56.4 ± 9.0 - -	56.6 ± 9.3 - -	56.2 ± 8.7 42 (31%) 92 (69%)	0.727
Anthropometric measurements				
Height (cm)	171.4 ± 9.7	177.8 ± 6.8	163.3 ± 6.3	<0.001
Weight (kg)	82.1 ± 16.5	88.9 ± 14.4	73.5 ± 15.0	<0.001
BMI (kg/m^2^)	27.9 ± 5.0	28.1 ± 4.5	27.6 ± 5.5	0.346
MRI measurements of the liver				
LIC (mg/g)	1.23 ± 0.12	1.26 ± 0.13	1.20 ± 0.10	<0.001
LFC (PDFF in %)	8.57 ± 7.9 5.61 (2.9–11.3)	10.35 ± 8.4 7.26 (4.1–14.0)	6.33 ± 6.4 3.85 (2.4–7.5)	<0.001 <0.001
Metabolic measurements				
Blood pressure				
Hypertension	107 (35.3%)	70 (41.4%)	37 (27.6%)	0.017
SBP (mmHg)	120.0 ± 16.5	125.3 ± 15.9	113.2 ± 14.6	<0.001
DBP (mmHg)	74.9 ± 10.0	77.1 ± 10.3	72.1 ± 8.7	<0.001
Glycemic Status				0.030
normoglycemic	190 (62.7%)	95 (56.2%)	95 (70.9%)	
prediabetes	76 (25.1%)	49 (29.0%)	27 (20.1%)	
diabetes	37 (12.2%)	25 (14.8%)	12 (9.0%)	
Behavior				
Physical Activity				0.028
no	76 (25.1%)	53 (31.4%)	23 (17.2%)	
sporadically	41 (13.5%)	21 (12.4%)	20 (14.9%)	
regularly around 1 h/week	97 (32.0%)	46 (27.2%)	51 (38.1%)	
regularly more than 2 h/week	89 (29.4%)	49 (29.0%)	40 (29.8%)	
Smoking				0.173
never-smoker	112 (37.0%)	56 (33.1%)	56 (41.8%)	
ex-Smoker	133 (43.9%)	82 (48.5%)	51 (38.1%)	
smoker	58 (19.1%)	31 (18.3%)	27 (20.1%)	
Medication				
antihypertensive	82 (27.1%)	47 (27.8%)	35 (26.1%)	0.842
lipid lowering	33 (10.9%)	17 (10.1%)	16 (11.9%)	0.737

Values are reported as arithmetic mean ± standard deviation (SD), *n* (%), or Median (IQR). *p*-values were derived from *t*-tests, *χ*^2^-tests, and Mann-Whitney-U tests, respectively. Hypertension: SBP > 140 mmHg and DBP > 90 mmHg, or receiving antihypertensive treatment, given that participant knew of hypertension. Glycemic status was defined according to WHO criteria: Normoglycemic: FBG < 110 mg/dL and 2 h-BG < 140 mg/dL. Prediabetes: normal FBG and 2 h-BG 140–200 mg/dL and/or FBG 110–125 mg/dL and normal 2 h-BG. Diabetes: FBG > 125 mg/dL and/or 2 h-BG > 200 mg/dL. Abbreviations: BMI body mass index, LIC liver iron content, LFC liver fat content, PDFF proton density fat fraction, SBP systolic blood pressure, DBP diastolic blood pressure, WHO World Health Organization, FBG fasting blood glucose, BG blood glucose.

**Table 2:** Distribution of mean daily food and nutrient intake.

	All	Men	Women	
	*N* = 303	*N* = 169	*N* = 134	*p*-value
Total energy intake (kcal/d)	1840 ± 414	2065 ± 352	1556 ± 295	<0.001
Macronutrients				
Carbohydrates (g/d)	192.9 ± 50.7	213.7 ± 49.5	166.5 ± 38.8	<0.001
Protein (g/d)	69.9 ± 15.0	76.6 ± 13.3	61.5 ± 12.5	<0.001
Fat (g/d)	77.1 ± 16.6	85.3 ± 14.2	66.6 ± 13.1	<0.001
Alcohol (Ethanol) (g/d)	11.7 ± 11.1	17.5 ± 11.3	4.4 ± 4.7	<0.001
Fiber (g/d)	16.5 ± 4.4	16.6 ± 4.5	16.3 ± 4.2	0.545
Sugars (g/d)	94.8 ± 32.6	101.3 ± 35.1	86.6 ± 27.1	<0.001
Saturated fatty acids (g/d)	34.8 ± 7.6	38.3 ± 6.7	30.3 ± 6.2	<0.001
Monounsaturated fatty acids (g/d)	27.3 ± 6.2	30.5 ± 5.2	23.2 ± 4.8	<0.001
Polyunsaturated fatty acids (g/d)	9.9 ± 2.6	10.8 ± 2.6	8.7 ± 2.0	<0.001
Omega-3-fatty acids (g/d)	1.5 ± 0.4	1.7 ± 0.4	1.3 ± 0.4	<0.001
Omega-6-fatty acids (g/d)	8.4 ± 2.2	9.2 ± 2.3	7.4 ± 1.7	<0.001
Omega-6: Omega-3 ratio	5.6 ± 1.0	5.6 ± 0.9	5.7 ± 1.1	0.843
Micronutrients				
Sodium (mg/d)	2119 ± 565	2390 ± 515	1777 ± 423	<0.001
Potassium (mg/d)	2537 ± 505	2653 ± 489	2390 ± 488	<0.001
Calcium (mg/d)	765.3 ± 206.6	745.9 ± 192.3	789.7 ± 221.7	0.067
Magnesium (mg/d)	284.7 ± 61.0	307.0 ± 59.1	256.6 ± 51.2	<0.001
Phosphorus (mg/d)	1111 ± 263	1201 ± 251	997 ± 234	<0.001
Chloride (mg/d)	3302 ± 808	3672 ± 727	2836 ± 648	<0.001
Iron (µg/d)	9647 ± 1931	10514 ± 1759	8553 ± 1548	<0.001
Zinc (µg/d)	9715 ± 2024	10603 ± 1731	8596 ± 1804	<0.001
Vitamin A—Retinol equivalent (µg/d)	1843 ± 648	1925 ± 656	1741 ± 625	0.014
Vitamin C—ascorbic acid (µg/d)	97,037 ± 28,481	92,328 ± 24,718	102,975 ± 31,724	0.002
Food groups				
Meat (g/d)	121.1 ± 42.5	145.5 ± 37.9	90.4 ± 24.3	<0.001
Red meat (g/d)	29.3 ± 12.3	35.4 ± 12.1	21.5 ± 7.1	<0.001
Cereal products (g/d)	166.2 ± 45.7	186.6 ± 41.7	140.5 ± 36.9	<0.001
Whole grain (g/d)	20.0 ± 18.6	20.1 ± 19.7	19.9 ± 17.2	0.949
Fruits and vegetables (g/d)	299.7 ± 108.9	268.9 ± 95.4	338.5 ± 112.8	<0.001
Fruits (g/d)	133.4 ± 69.3	122.6 ± 68.3	147.1 ± 68.4	0.002
Vegetables (g/d)	166.3 ± 60.8	146.3 ± 45.7	191.4 ± 68.0	<0.001
Dairy products (g/d)	186.4 ± 103.8	172.1 ± 102.7	204.4 ± 102.7	0.007

Values are reported as arithmetic mean ± SD and *p*-values were derived from *t*-tests. Abbreviations: /d: per day.

**Table 3:** Association of food and nutrient intake with LIC in men and women.

	LIC (µg/g) in Men (*N* = 169)			LIC (µg/g) in Women (*N* = 134)		
	β & CI	*p*-Value	Adj. R^2^	β & CI	*p*-Value	Adj. R^2^
Carbohydrates (g/d)	−0.590 (−1.413; 0.233)	0.159	0.103	***−0.970 (−1.992; 0.053)***	***0.063***	***0.355***
Protein (g/d)	−0.494 (−2.97; 1.987)	0.695	0.093	−1.040 (−3.239; 1.159)	0.351	0.342
Fat (g/d)	−0.049 (−2.425; 2.326)	0.967	0.092	0.315 (−2.186; 2.816)	0.804	0.338
Alcohol (Ethanol) (g/d)	**1.698 (0.002; 3.394)**	**0.050**	**0.113**	**5.615 (2.687; 8.543)**	**<0.001**	**0.404**
Fiber (g/d)	−1.789 (−7.233; 3.654)	0.517	0.094	**−5.818 (−10.835; −0.801)**	**0.023**	**0.364**
Sugars (mg/d)	−0.000 (−0.001; 0.000)	0.165	0.103	−0.000 (−0.001; 0.000)	0.460	0.340
Saturated fatty acids (mg/d)	−0.002 (−0.007; 0.003)	0.385	0.096	−0.001 (−0.006; 0.003)	0.583	0.339
Monounsaturated fatty acids (mg/d)	0.002 (−0.004; 0.007)	0.554	0.094	0.003 (−0.003; 0.008)	0.381	0.341
Polyunsaturated fatty acids (mg/d)	0.002 (−0.007; 0.012)	0.653	0.093	0.002 (−0.009; 0.013)	0.711	0.338
Omega-3-fatty acids (mg/d)	0.019 (−0.038; 0.076)	0.509	0.095	0.001 (−0.039; 0.041)	0.949	0.338
Omega-6-fatty acids (mg/d)	0.002 (−0.008; 0.012)	0.711	0.093	0.002 (−0.010; 0.015)	0.691	0.338
Omega-6: Omega-3 ratio	−0.604 (−25.471; 0.134)	0.540	0.094	2.423 (−10.513; 1.536)	0.712	0.338
Sodium (mg/d)	−0.018 (−0.067; 0.032)	0.487	0.095	−0.007 (−0.058; 0.044)	0.781	0.338
Potassium (mg/d)	0.018 (−0.042; 0.079)	0.547	0.094	**−0.058 (−0.111; −0.005)**	**0.034**	**0.360**
Calcium (mg/d)	−0.018 (−0.148; 0.112)	0.781	0.093	−0.014 (−0.118; 0.091)	0.793	0.338
Magnesium (mg/d)	0.186 (−0.338; 0.709)	0.484	0.095	***−0.505 (−1.055; 0.045)***	***0.071***	***0.354***
Phosphorus (mg/d)	0.023 (−0.116; 0.163)	0.743	0.093	−0.059 (−0.182; 0.064)	0.344	0.342
Chloride (mg/d)	−0.007 (−0.043; 0.029)	0.685	0.093	−0.006 (−0.043; 0.030)	0.729	0.338
Iron (µg/d)	0.004 (−0.014; 0.023)	0.632	0.093	−0.011 (−0.027; 0.006)	0.198	0.346
Zinc (µg/d)	−0.003 (−0.022; 0.016)	0.761	0.093	−0.006 (−0.021; 0.009)	0.441	0.341
Vitamin A-Retinol equivalent (µg/d)	0.003 (−0.028; 0.034)	0.837	0.092	−0.020 (−0.046; 0.005)	0.119	0.350
Vitamin C—ascorbic acid(µg/d)	−0.000 (−0.001; 0.001)	0.808	0.092	−0.000 (−0.001; 0.000)	0.485	0.340
Meat (g/d)	−0.152 (−0.713; 0.410)	0.594	0.094	−0.203 (−0.882; 0.475)	0.555	0.339
Red meat (g/d)	−0.006 (−1.631; 1.516)	0.942	0.092	0.280 (−1.856; 2.417)	0.796	0.338
Cereal products (g/d)	−0.020 (−0.633; 0.592)	0.948	0.092	−0.310 (−0.84; 0.23)	0.258	0.344
Whole grain (g/d)	−0.184 (−1.189; 0.822)	0.719	0.093	−0.585 (−1.267; 0.296)	0.191	0.346
Fruits and vegetables (g/d)	−0.018 (−0.229; 0.193)	0.868	0.092	−0.097 (−0.239; 0.045)	0.177	0.347
Fruits (g/d)	−0.217 (−0.503; 0.070)	0.137	0.104	−0.179 (−0.403; 0.044)	0.114	0.350
Vegetables (g/d)	***0.420 (−0.011; 0.852)***	***0.056***	***0.112***	−0.070 (−0.307; 0.168)	0.563	0.339
Dairy products (g/d)	−0.068 (−0.264; 0.128)	0.494	0.095	−0.123 (−0.292; 0.045)	0.150	0.348

Presented are results from a linear regression model with outcome LIC in µg/g dry weight and the respective nutrition variable as exposure of interest (respective unit in table). Models were adjusted for age (years), energy intake (kcal/day), and log-transformed LFC (log-PDFF). Estimates that reached **significance** or ***marginal significance***, are presented in **bold** and ***bold italics***, respectively. Abbreviations: *β* beta coefficient, CI confidence interval, Adj. R^2^ adjusted R-squared (proportion of variance in the outcome explained by independent variables), LIC liver iron content, LFC liver fat content, /d: per day.

## Data Availability

The informed consent given by KORA study participants does not cover data posting in public databases. However, data are available upon request by means of a project agreement. Requests should be sent to kora.passt@helmholtz-muenchen.de and are subject to approval by the KORA Board.

## Supplementary Materials

The following are available online at https://www.mdpi.com/article/10.3390/nu14010132/s1, Figure S1: Distribution of liver iron content per menopausal status; Table S1: Summary statistics for liver iron in the units [1/s] and [mg/g] based on available publications; Table S2: Baseline characteristics of women according to menopausal status. Table S3: Comparison of baseline characteristics for included and excluded participants.
