# Supplementary material for: Association of Habitual Dietary Intake with Liver Iron—A Population-Based Imaging Study
Source: Nutrients. 2021 Dec 28;14(1):132. doi: 10.3390/nu14010132 (PMC8746950; doi:10.3390/nu14010132)
Supplement: Supplementary file 1 [file nutrients-14-00132-s001.zip › nutrients-1504977-supplementary.pdf]

Supplementary Materials to
Association of habitual dietary intake with liver iron – a population-based imaging study

Jule Filler[1,2,3], Ricarda von Krüchten[4], Nina Wawro[5,6], Lisa Maier[1,2,3], Roberto Lorbeer[7,8], Johanna Nattenmüller[4,9], Barbara Thorand[1,10], Fabian Bamberg[4], Annette Peters[1,2,10], Christopher L. Schlett[4], Jakob Linseisen[2,5,6], Susanne Rospleszcz[2,1]*

[1] Institute of Epidemiology, Helmholtz Zentrum München, Ingolstädter Landstraße 1, 85764, Neuherberg, Germany
[2] Institute for Medical Information Processing, Biometry and Epidemiology – IBE, LMU Munich, Munich, Germany.
[3] Pettenkofer School of Public Health, Munich, Germany.
[4] Department of Diagnostic and Interventional Radiology, Medical Center, University Freiburg, Freiburg, Germany.
[5] Independent Research Group Clinical Epidemiology, Helmholtz Zentrum München, German Research Center for Environmental Health (GmbH), Neuherberg, Germany.
[6] Chair of Epidemiology, University Augsburg, at University Hospital Augsburg, Augsburg, Germany.
[7] Department of Radiology, University Hospital, LMU Munich, Munich, Germany.
[8] German Centre for Cardiovascular Research (DZHK e.V.), Munich, Germany.
[9] Clinic of Diagnostic and Interventional Radiology (DIR), Heidelberg University Hospital, Heidelberg, Germany.
[10] German Center for Diabetes Research (DZD), München-Neuherberg, Germany.

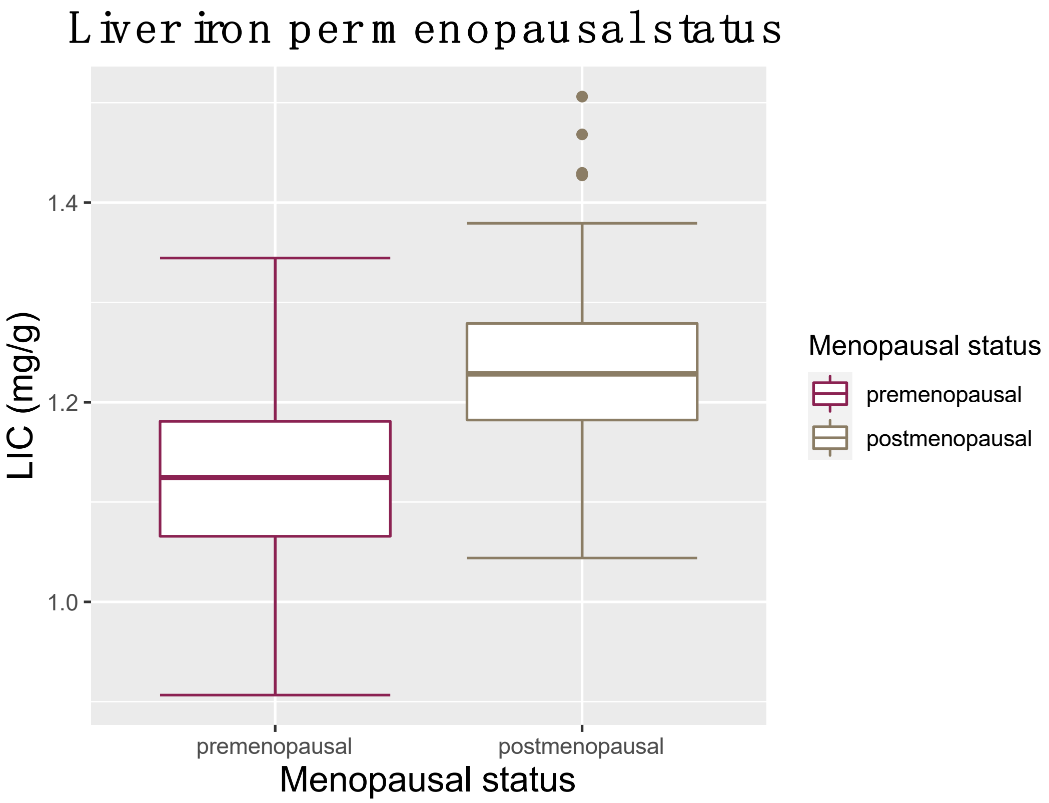

Figure S1. Distribution of liver iron content (LIC) per menopausal status. Comparison of 42 (31%) premenopausal and 92 (69%) postmenopausal women. Upper and lower bound of the box represent 1st and 3rd Quartile (Interquartile Range, IQR), thick horizontal lines, the median. Whiskers show maximum or minimum value of the data that is within 1.5 times the IQR over or under the 1st or 3rd Quartile, respectively. Extreme values (over 1.5 times IQR under/over 1st/3rd Quartile) are depicted as dots.

Supplemental Table S1. Summary statistics for liver iron in the units [1/s] and [mg/g] based on available publications.

Unit	1/s	mg/g						
Publication		Anderson 2001 (1)	Hankins 2009 (2)	Garbowski 2014 (3)	Wood 2005 (4)	Paisant 2017 (5)	Henninger 2015 (6)	Henninger 2017 (7)
**Mean**	40.54	0.32	0.68	1.16	1.23	1.25	1.25	1.56
**SD**	4.71	0.07	0.13	0.15	0.12	0.14	0.11	0.15
**1st Quartile**	37.62	0.28	0.60	1.06	1.16	1.17	1.18	1.47
**Median**	40.42	0.32	0.68	1.15	1.23	1.25	1.25	1.56
**3rd Quartile**	43.46	0.36	0.77	1.25	1.31	1.33	1.32	1.65
**p-value Wood vs. X**		<0.001	<0.001	<0.001		0.079	0.054	<0.001

All of the cited intervention studies compared MRI-derived liver iron values and liver-biopsy derived iron concentration in populations of 17-54 subjects (1-7). Four of them additionally performed MRI on samples of up to 106 subjects (1, 3, 4, 6). Participants with elevated iron concentrations due to underlying clinical conditions such as transfusional hemosiderosis, thalassemia major, or sickle cell anemia were included. The present study used the formula specified by Wood et al. (8) for liver iron unit conversion (shaded in grey). The structure of this table as well as presented publications are based on Table S2 from McKay et al. (8).

Supplemental Table S2. *Comparison of baseline characteristics for pre- and postmenopausal women.*

	Premenopausal	Postmenopausal	
	N = 42	N = 92	p-value
Age (years)	46.9 ± 4.5	60.4 ± 6.6	< 0.001
BMI (kg/m^2)	26.7 ± 6.4	28.0 ± 5.0	0.237
**Metabolic measurements**			
Blood pressure			
Hypertension	5 (11.9%)	32 (34.8%)	0.011
SBP (mmHg)	109.2 ± 12.3	115.0 ± 15.2	0.032
DBP (mmHg)	71.1 ± 7.9	72.5 ± 9.1	0.375
Glycemic Status			0.210
normoglycemic	34 (80.6%)	61 (66.3%)	
prediabetes	6 (14.3%)	21 (22.8%)	
diabetes	2 (4.8%)	10 (10.9%)	
**Behavior**			
Physical Activity			0.216
no	4 (9.5%)	19 (20.7%)	
sporadically	9 (21.4%)	11 (12.0%)	
regularly around 1h/week	18 (42.9%)	33 (35.9%)	
regularly more than 2h/week	11 (26.2%)	29 (31.5%)	
Smoking			0.497
never-smoker	16 (38.1%)	40 (43.5%)	
ex-smoker	19 (45.2%)	32 (34.8%)	
smoker	7 (16.7%)	20 (21.7%)	
**Medication**			
antihypertensive	4 (9.5%)	31 (33.7%)	0.006
lipid lowering	1 (2.4%)	15 (16.3%)	0.044
**MRI-derived values**			
LIC (mg/g)	1.13 ± 0.09	1.23 ± 0.09	< 0.001
LFC (PDFF, %)	3.6% ± 3.2	7.6 % ± 7.1	< 0.001

Values are reported as arithmetic mean ± standard deviation (SD) or n (%). P-values were derived from t-test and $\chi2$- tests, respectively; Hypertension was defined as SBP > 140 mmHg and DBP > 90 mmHg, or receiving antihypertensive treatment, given that participant knew of hypertension; Glycemic status was defined according to WHO criteria (9):. Normoglycemic: FBG < 110 mg/dL and 2h-BG < 140 mg/dL; Prediabetes: normal FBG and 2h-BG 140-200 mg/dL and/or FBG 110-125 mg/dL and normal 2h- BG; Diabetes: FBG > 125 mg/dL and/or 2h-BG > 200 mg/dL; Abbreviations: BMI body mass index, LIC Liver Iron Content, LFC Liver Fat Content, PDFF proton density fat fraction, SBP systolic blood pressure, DBP diastolic blood pressure, WHO World Health Organization, FBG fasting blood glucose, BG blood glucose.

nutrients MDPI

Supplemental Table S3. Comparison of baseline characteristics for included and excluded participants.

	Included	Excluded	
	N = 303	N = 97	p-value
Sex	169 male (55.79%)	62 male (63.9%)	0.200
Age (years)	56.4 ± 9.0	56.0 ± 9.7	0.720
BMI (kg/m^2)	27.9 ± 5.0	28.8 ± 4.7	0.122
**Metabolic measurements**			
Blood pressure			
Hypertension	107 (35.3%)	29 (29.9%)	0.391
SBP (mmHg)	120.0 ± 16.5	122.6 ± 17.6	0.186
DBP (mmHg)	74.9 ± 10.0	76.4 ± 10.1	0.180
Glycemic Status			0.281
normoglycemic	190 (62.7%)	53 (54.6%)	
prediabetes	76 (25.1%)	27 (27.8%)	
diabetes	37 (12.2%)	17 (17.5%)	
**Behavior**			
Physical Activity			0.590
no	76 (25.1%)	29 (29.9%)	
sporadically	41 (13.5%)	16 (16.5%)	
regularly around 1h/week	97 (32.0%)	26 (26.8%)	
regularly more than 2h/week	89 (29.4%)	26 (26.8%)	
Smoking			0.749
never-smoker	112 (37.0%)	34 (35.0%)	
ex-smoker	133 (43.9%)	41 (42.3%)	
smoker	58 (19.1%)	22 (22.7%)	
**Medication**			
antihypertensive	82 (27.1%)	20 (20.6%)	0.257
lipid lowering	33 (10.9%)	10 (10.3%)	1.000
**MRI-derived values**			
LIC (mg/g)*	1.23	1.24	0.546
LFC (PDFF, %)†	8.6% ± 7.9	9.7% ± 8.6	0.252

* analysis was performed on n = 83 excluded patients with available LIC data; † analysis was performed on n = 86 excluded patients with available LFC data; Values are reported as arithmetic mean ± standard deviation (SD) or n (%). LFC distribution is also described by median and interquartile range. P-values were derived from t-test, χ^2- tests, and Mann-Whitney-U tests, respectively; Hypertension was defined as SBP > 140 mmHg and DBP > 90 mmHg, or receiving antihypertensive treatment, given that participant knew of hypertension; Glycemic status was defined according to WHO criteria (9): ; Normoglycemic: FBG < 110 mg/dL and 2h-BG < 140 mg/dL; Prediabetes: normal FBG and 2h-BG 140-200 mg/dL and/or FBG 110-125 mg/dL and normal 2h- BG; Diabetes: FBG > 125 mg/dL and/or 2h-BG > 200 mg/dL; Abbreviations: BMI body mass index, LIC Liver Iron Content, LFC Liver Fat Content, PDFF proton density fat fraction, SBP systolic blood pressure, DBP diastolic blood pressure, WHO World Health Organization, FBG fasting blood glucose, BG blood glucose.

References

1. Anderson, L.J., S. Holden, B. Davis, E. Prescott, C.C. Charrier, N.H. Bunce, D.N. Firmin, B. Wonke, J. Porter, J.M. Walker; et al., *Cardiovascular T2-star (T2*) magnetic resonance for the early diagnosis of myocardial iron overload.* Eur Heart J, **2001**, *22*, 2171–2179.
2. Hankins, J.S., M.B. McCarville, R.B. Loeffler, M.P. Smeltzer, M. Onciu, F.A. Hoffer, C.S. Li, W.C. Wang, R.E. Ware, and C.M. Hillenbrand, *R2* magnetic resonance imaging of the liver in patients with iron overload.* Blood, **2009**, *113*, 4853–4855.
3. Garbowski, M.W., J.P. Carpenter, G. Smith, M. Roughton, M.H. Alam, T. He, D.J. Pennell, and J.B. Porter, *Biopsy-based calibration of T2* magnetic resonance for estimation of liver iron concentration and comparison with R2 Ferriscan.* J Cardiovasc Magn Reson, **2014**, *16*, 40.
4. Wood, J.C., C. Enriquez, N. Ghugre, J.M. Tyzka, S. Carson, M.D. Nelson, and T.D. Coates, *MRI R2 and R2* mapping accurately estimates hepatic iron concentration in transfusion-dependent thalassemia and sickle cell disease patients.* Blood, **2005**, *106*, 1460–1465.
5. Paisant, A., G. d'Assignies, E. Bannier, E. Bardou-Jacquet, and Y. Gandon, *MRI for the measurement of liver iron content, and for the diagnosis and follow-up of iron overload disorders.* Presse Med, **2017**. *46*, e279-e287.
6. Henninger, B., H. Zoller, S. Rauch, A. Finkenstedt, M. Schocke, W. Jaschke, and C. Kremser, *R2* relaxometry for the quantification of hepatic iron overload: Biopsy-based calibration and comparison with the literature.* Rofo, **2015**, *187*, 472–479.
7. Henninger, B., H. Zoller, S. Kannengiesser, X. Zhong, W. Jaschke, and C. Kremser, *3D Multiecho Dixon for the Evaluation of Hepatic Iron and Fat in a Clinical Setting.* J Magn Reson Imaging, **2017**, *46*, 793–800.
8. McKay, A., H.R. Wilman, A. Dennis, M. Kelly, M.L. Gyngell, S. Neubauer, J.D. Bell, R. Banerjee, and E.L. Thomas, *Measurement of liver iron by magnetic resonance imaging in the UK Biobank population. PLoS ONE*, **2018**, *13*, e0209340.
